# The Total Mastectomy and Subsequent Replacement of Breasts in the Mouse

**DOI:** 10.1038/bjc.1965.20

**Published:** 1965-03

**Authors:** J. M. Riggott

## Abstract

**Images:**


### THE TOTAL MASTECTOMY AND SUBSEQUENT REPLACEMENT OF BREASTS IN THE MOUSE

J. M. RIGGOTT*

*From the Cancer Research Laboratories, Department of Pathology, The Medical School, Birmingham*

Received for publication October 8, 1964

### MASTECTOMY OF THE MOUSE

THE total removal of breasts from small laboratory animals was first described by Long and McLean (1922). They attempted the removal of the breasts from 8-day-old rats, but succeeded in only a small number of cases. Fekete (1939) studied the development of the breasts in *dba* and C57Bl mice, before attempting the total removal of the breasts from 10-day-old mice by extirpation of the fat pads in the region of each nipple. There was a very high mortality rate in the operated animals. Amongst the survivors she reported a 75 per cent breast tumour incidence after their infection with mammary tumour agent (MTA). Faulkin and DeOme (1960) described a method for the removal of the 4th breast in 3-week-old female mice of the C3H strain. They made an inverted " Y "-shaped incision along the abdominal midline and laterally between the 4th and 5th nipples midway down each hind leg. Then, by pinning back the skin flaps so obtained, they exposed the 4th breasts and, after cauterisation of the main blood vessels, dissected them out of their fat pads. Dux (1962) described a method for the ablation of the breasts in female rats and mice, which was similar in many respects to that of Faulkin and DeOme (1960) but extends their technique to incorporate all 10 breasts.

The methods for the total removal of breasts so far reported made no attempt to assess immediately to completeness of the operation. It was decided to devise a technique for the removal of all 10 breasts from mice and to attempt to devise a method to assess the completeness of the operation without killing or interfering further with operated animals.

A preliminary study of the growth and development of the breasts in young mice showed that an intensive growth phase occurred at $3\frac{1}{2}$ weeks from birth in the breasts of $F_1$ hybrid (C57Bl $\times$ IF) mice, and later in the parent strains. The optimum age for the total removal of breasts in the hybrids which were used in this experiment was judged to be immediately before this, at exactly 3 weeks from birth. The mice would then be large enough to be weaned and this would avoid the danger of their being killed by their mother after the operation.

The direction and extent of growth of the breasts at 3 weeks old (Fig. 1) was determined by stripping off the whole skin, staining with Mayer's haemalum by the method described later, and examining under the dissecting microscope.

* Present address: Department of Zoology, The University, Hull, East Yorkshire.

*Operation procedure*

*Preparation of the operation site.*—The mice were anaesthetised by intraperitoneal injections of sodium nembutal (Abbott) prepared and administered at 0·06 mg. per g. body weight, according to the method described by Pilgrim and DeOme (1955).

Each 3-weeks-old hybrid female was stripped of its fur on the ventral surface, from the neck down as far as the vagina and to the mid-lateral line on each side, using a 3 : 1 starch-barium sulphide mixture, made into a thin paste with warm water. The depilatory paste and fur were removed after about 3 minutes by

Fig. 1.—Diagram of the underside of the pelt of a 3-week-old female hybrid to show the amount and direction of growth of the breasts in their fat pads.

washing in warm water. This exposed all the nipples, making them clearly visible under the dissecting microscope.

*Removal of the nipples.*—The animal was placed ventral side uppermost on an electrically-heated operating table, maintained at 94° F. The skin surface was wiped liberally with 5 per cent " Cetavlon " (I.C.I. Ltd., Pharmaceuticals Division, Cheshire) in 70 per cent spirit. Each nipple, in turn, was held in fine-pointed watch-maker's forceps and a circle 5 mm. in diameter was cut with curved iridectomy scissors in the skin round it. The main duct of the breast to the nipple could then be seen by lifting the skin circle bearing the nipple. This duct was cut as close to the nipple as possible, to prevent damage to the underlying breast, and the excised nipple with skin circle was discarded.

*Removal of the fat pad containing the breast tissue.*—The exposed fat pad was grasped firmly with watch-makers forceps and dissected from the skin and underlying muscles. A large amount of each fat pad was excised, as shown in Fig. 2, to ensure the complete removal of the breast tissue. Superficial bleeding occurred during the operation, but clotting followed quickly and little swabbing was necessary.

*Post operative care.*—The mastectomised animal was powdered liberally with sulphadiazine to aid haemostasis and prevent infection. No sutures were

FIG. 2.—Diagram to show the stages of the mastectomy operation. ex. s. = The piece of skin excised for the removal of the nipple. ex. fp. = Dotted outline of part of the fat pad which is excised.

necessary. It was allowed to recover in a warm place. A small number of animals died due to too much heat, the heat from a 40 watt lamp about 1 foot above the animals was found to be adequate.

Scabs formed over the wounds within 3 days and at no time did the animals suffer inconvenience in feeding or drinking.

*Histological preparation and examination of excised breast tissue.*—During the operation each excised fat pad was teased out on an alcohol/ether cleaned slide. The 5 breasts from the right side of the animal were placed in order on one slide and the 5 from the left side on another (Fig. 3A) to simplify the subsequent identification of the glands. The slides were then dipped in 5 per cent aqueous gelatin and fixed overnight in 25 per cent formalin. This treatment prevented the excised fat pads from washing off the slides during subsequent histological procedures.

This tissue was dehydrated through 50, 70, 90 per cent and absolute alcohol, with 2 hours in each solution, and the fat removed by soaking in chloroform overnight. The chloroform was removed by 2 half-hourly washes in absolute alcohol and the tissue re-hydrated through 90, 70 and 50 per cent alcohol and then in water overnight. The re-hydrated breast tissue was stained for 2 minutes in Mayer's (1903) haemalum and blued in running water. The tissue was examined in this state under the dissecting microscope and any small strips of muscle masking the breasts were dissected off. The resulting preparations were unevenly stained, so they were then decolorised in acid alcohol, washed in running water and restained for 6 minutes in Mayer's haemalum. After blueing for 10 minutes in running water, they were then dehydrated in alcohol as before, soaked for $\frac{1}{4}$ hour in carbol-xylol, cleared in xylol for 10 minutes and mounted in neutral canada balsam.

The slides were dried in a 57° C. incubator overnight, after which they were examined microscopically.

Fig. 3B shows a completely excised breast. Any animal whose prepared breast tissue was found on examination to consist of 10 breasts similar to the one illustrated was assumed to be completely mastectomised.

*Estimation of the efficiency of the technique*

Twelve female hybrids were mastectomised at 3 weeks old, and the excised breasts prepared and examined as described above. The presence of complete or incomplete breasts in the preparations obtained was scored against each mouse. It was judged that 7 of the 12 mice were completely mastectomised and it was suspected that each of the other 5 retained one breast remnant per mouse. When the 12 mice were 2 months old, they were all mated in order to ensure maximum development of any breast remnants. They were killed at full term. The fur was completely removed with the depilatory paste described above. The skin was removed by a mid-dorsal incision from the head to the base of the tail and careful dissection of the remains of the fat pads from the underlying muscles. The skins so obtained, bearing the remains of the fat pads, were pinned out on a cork board and fixed overnight in formol-saline. They were dehydrated slowly through alcohols as previously described and soaked in chloroform for 3 days to remove all

EXPLANATION OF PLATE

FIG. 3.—A. A slide of 5 excised breasts in part of their fat pads, from a 3-week-old hybrid female—stained in Mayer's haemalum. ×6. B. A complete, excised 5th breast from a 3-week-old hybrid female. Stained in Mayer's haemalum. ×18. C. A supernumerary breast from the 5th region of a female hybrid at full term pregnancy. Stained in Mayer's haemalum. ×6. D. Breast tissue 6 months after grafting to breast-free hybrid host. Stained in Mayer's haemalum. ×9.

Riggott.

the fat. After hydration they were stained in Mayer's haemalum for 6 minutes and blued for $\frac{1}{2}$ hour in running water. The skins were then dehydrated in alcohol, soaked in carbol-xylol and cleared in xylol. They were examined under the dissecting microscope for the presence of breast remnants. The results are compared with those obtained by examination of the histological preparations of the excised breast tissue in Table I.

TABLE I.—*The Estimation of Complete Mastectomy by Comparison of the Results Obtained from Examination of Excised Breasts, with* post mortem *Examination of Prepared Skins*

| Examination of excised breasts | | |
|---|---|---|
| No. of mice examined | No. of mice judged completely mastectomised | No. of mice believed incompletely mastectomised |
| 12 | 7 | 5 |
| *Post mortem* examination of prepared skins | | |
| No. of skins examined | No. of skins without breast remnants | No. of skins with breast remnants |
| 12 | 9 | 3 |

The *post mortem* examination showed that breast tissue developed in 3 mice. Only one of these 3 mice had been suspected of being incompletely mastectomised from the examination of the excised breasts, the remaining 4 suspects proving, in fact, to be devoid of breast remnants.

The other 2 mice which had developed breast tissue, on *post mortem* examination, were 2 of the 7 which were believed to be completely mastectomised. These 2 mice developed breast tissue in the region of the 5th fat pad. One of these deposits is shown in Fig. 3C. The 5th breast removed from this mouse is shown in Fig. 3B and is seen to be complete.

### DISCUSSION

The only explanation for the growth of breast tissue in the last 2 mice would seem to be that in some small proportion of animals supernumerary breasts occur in the 5th region. The presence of supernumerary nipples in this region has never been observed. These supernumerary breasts would therefore be overlooked by the technique described.

Dux (1962) found that supernumerary breasts occurred in the region of the 5th breast in some of the mouse strains she examined. Fekete (1939) also had trouble, particularly in the 5th region, with her attempted mastectomy. It would seem that in order to ensure complete mastectomy in every mouse, it would be necessary to remove all subcutaneous fat in the area round the vagina and anus.

If mice mastectomised by the above technique are to be used as hosts for the implantation of breast tissue grafts it is possible to remove from them any breast remnants detected by the examination of excised tissue by a further operation within 7 days. This means opening the animal again in the region where the remnant is suspected and removing the remaining fat pad of that particular breast. Breast tissue grafting can be carried out at the same time.

METHOD OF IMPLANTATION OF BREAST TISSUE GRAFTS

*Operation procedure*

*Preparation of breast grafts.*—The donor animal was prepared in the same way as described for the mastectomy operation. In turn each nipple was excised and discarded. All 10 breasts were excised and left under the cut skin of the donor animal in their original sites until required for transplantation. The method of excision being the same as that described for the mastectomy operation.

*Grafting of the mastectomised hosts.*—This operation was carried out about 7 days after mastectomy. The sites for implantation were chosen lateral to the original wounds since the wounds from the first operation were incompletely healed and to open them again would have presented difficulty in subsequent suturing,

FIG. 4.—Diagram of the left side of a mouse to show the position of the incisions into which the implanted breasts were inserted. The direction of insertion is indicated by the arrows. The numbers refer to the implanted breasts (c.f. Fig. 1).

due to the large deposits of early scar and granulation tissue. This placed the implanted breasts in juxtaposition with the remains of the host's own fat pads.

Under nembutal anaesthesia the skin of the mastectomised animal was swabbed with " Cetavlon ". A small incision was made at one of the positions indicated in Fig. 4.

The skin was gently dissected free from the subcutaneous tissue and the fat pad to be implanted was pushed into the wound in the direction indicated by the arrows in Fig. 4. The skin was sutured with two small stitches of Chinese silk and, after implantation of 10 fat pads, the animals were allowed to recover in the warm.

*Fate of grafted breast tissue.*—In animals which were killed 6 months after the implantation of 10 breasts, it was possible to trace the fate of all the grafted breasts by examination of preparations of whole skins. (The method of preparation of the skin is described above.)

The implanted breasts still retained their original rounded shape, all were well vascularised and the breast tissue had filled out the implanted fat (Fig. 3D). In the majority of cases the breast tissue had grown out of the implants and colonised the remains of the hosts own fat pads, this occurred especially in the thoracic region.

### DISCUSSION

The present results show that, when implanted under the skin of genetically similar mastectomised hosts, immature breast tissue will grow to fill out the fat pad in which it was originally transplanted. It is also capable of colonising remnants of host fat pads with which it may come into contact.

The techniques of mastectomy and breast implantation described here provide a means of complete replacement of the breast tissue of an immature mouse by breast tissue from an immunologically compatible immature donor. The removal of the 10 breasts of any individual mouse can be assessed by histological examination of the tissue removed. The presence of supernumerary breasts in the animals used here indicates the necessity for a preliminary search for these to be made in each genetic type of mouse contemplated for the mastectomy operation. Special precautions would be required to remove these supernumeraries and thereby ensure the complete mastectomy of every mouse.

It has been shown that breast replacement can be performed by subcutaneous implantation of fat pads containing breast tissue from an immature animal. If adult mice were required as donors of breast tissue it should be possible to make large skin grafts bearing breast tissue, as described by Prehn (1953).

These techniques make possible a study of the intrinsic properties of the breast tissue itself when exposed to different host environments. Their use in an investigation of the part played by intrinsic properties of the breast tissue in breast carcinogenesis by methylcholanthrene will be described in a subsequent paper (Riggott, 1965).

### SUMMARY

A method has been described for the total removal of breast tissue from 3-week-old female mice. The degree of mastectomy was assessed by histological examination of the excised tissue from each individual mouse. This proved successful except in a few cases where supernumerary breasts were present.

Mastectomised mice have been grafted subcutaneously with 10 fat pads bearing breasts from 3-week-old donors of the same genetic type. The implanted breasts grew to fill the donor fat pad and in many cases colonised remains of their host's own fat pads.

This work formed part of a thesis for the degree of Ph.D. in the Faculty of Medicine of the University of Birmingham. I wish to express my thanks to Dr. June Marchant for her help during the course of this work.

I am grateful to the Birmingham Branch of the British Empire Cancer Campaign for Research for the support of this work.

### REFERENCES

Dux, A.—(1962) *Nature, Lond.*, **196**, 287.
Faulkin, L. J. Jr. and DeOme, K. B.—(1960) *J. nat. Cancer Inst.*, **24**, 953.
Fekete, E.—(1939) *Anat. Rec.*, **73**, 319.
Long, J. A. and McLean, H. E.—(1922) *Mem. Univ. Calif.*, **6**, 1.
Mayer, P.—(1903) *Z. wiss. Mikr.*, **20**, 409.
Pilgrim, H. I. and DeOme, K. B.—(1955) *Exp. Med. Surg.*, **13**, 401.
Prehn, R. T.—(1953) *J. nat. Cancer Inst.*, **13**, 859.
Riggott, J. M.—(1965) *Brit. J. Cancer*, **19**, 174.


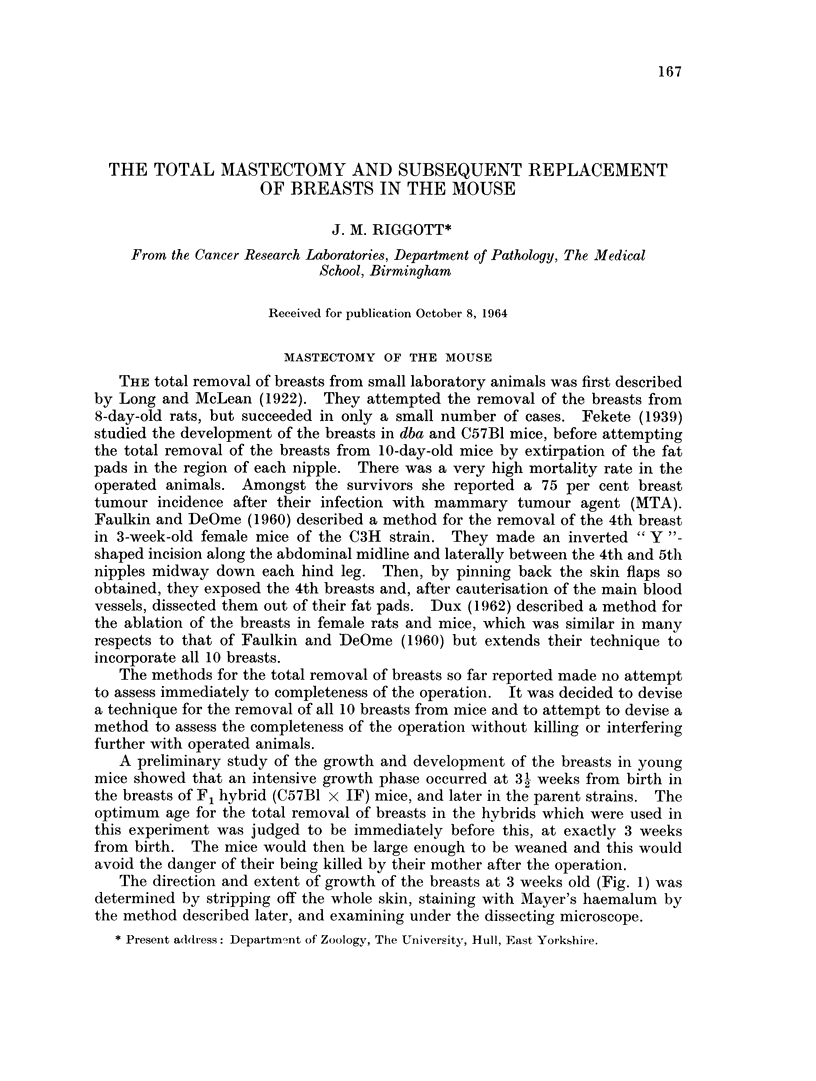

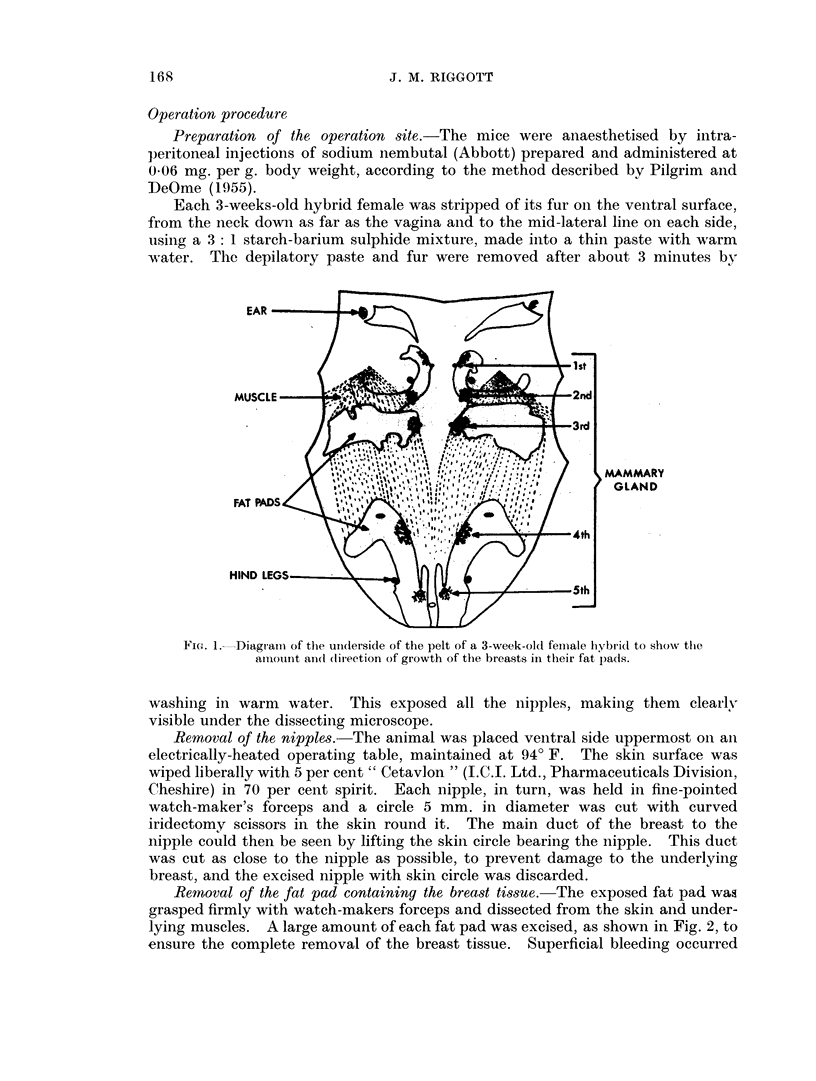

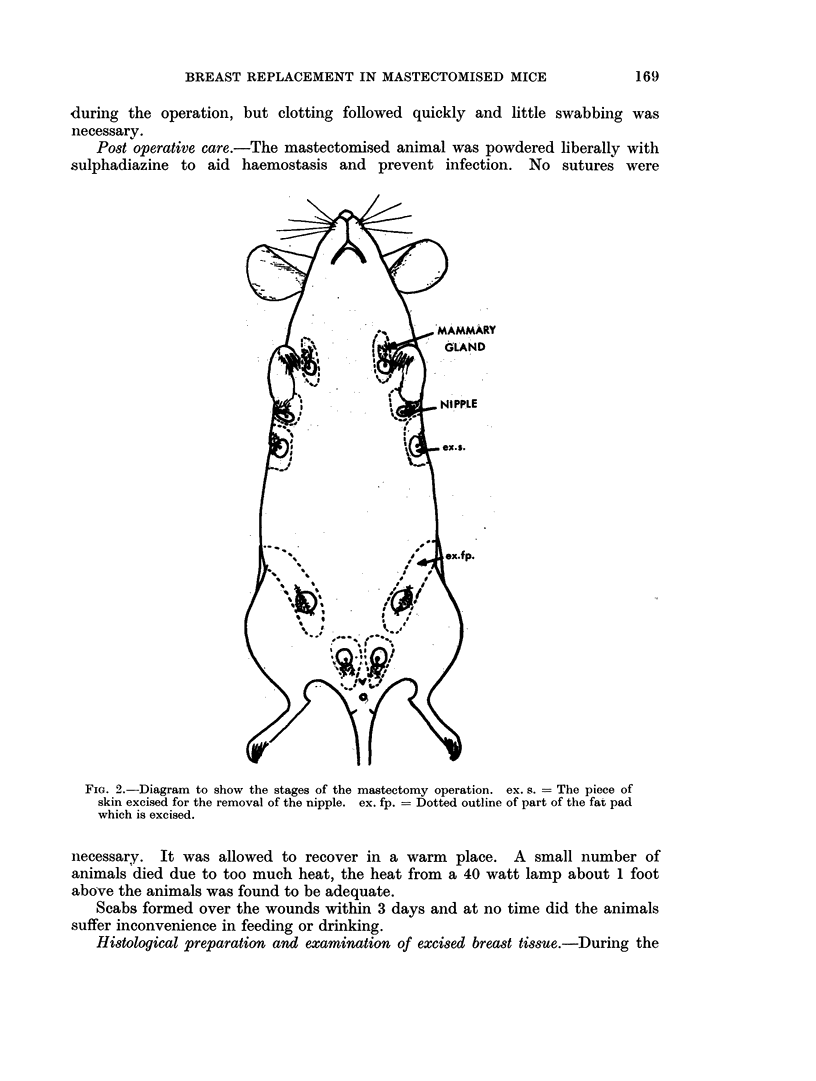

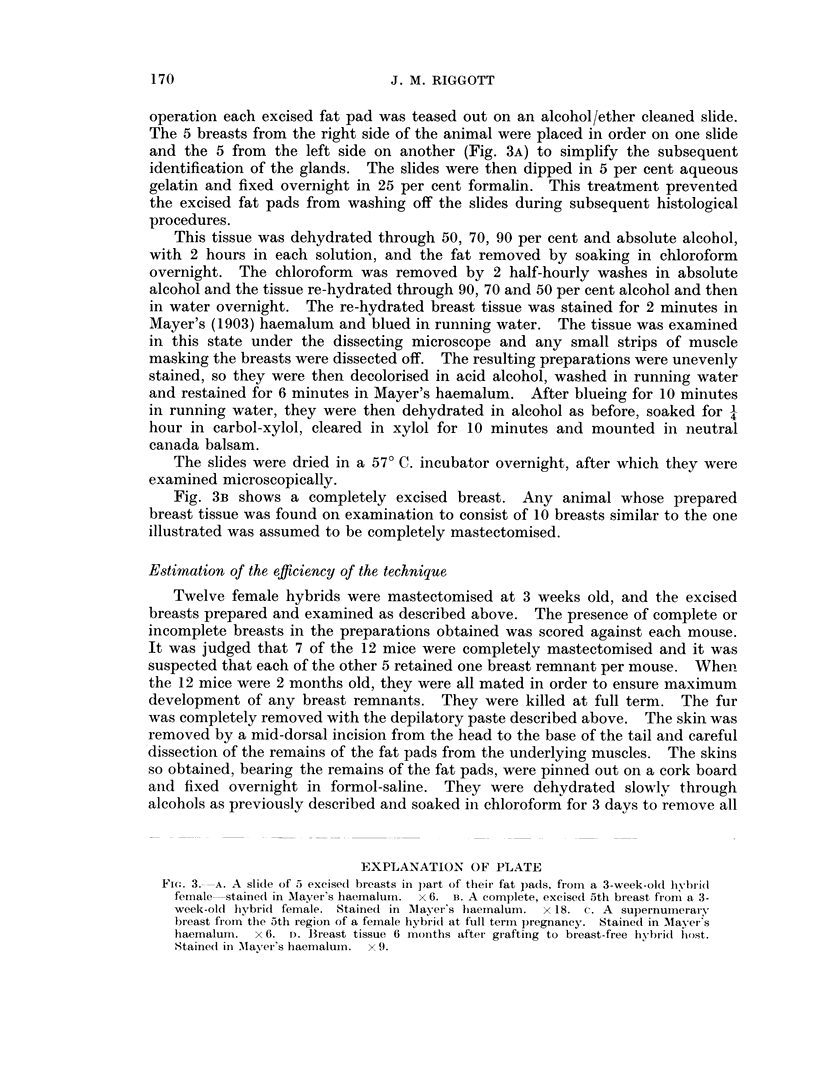

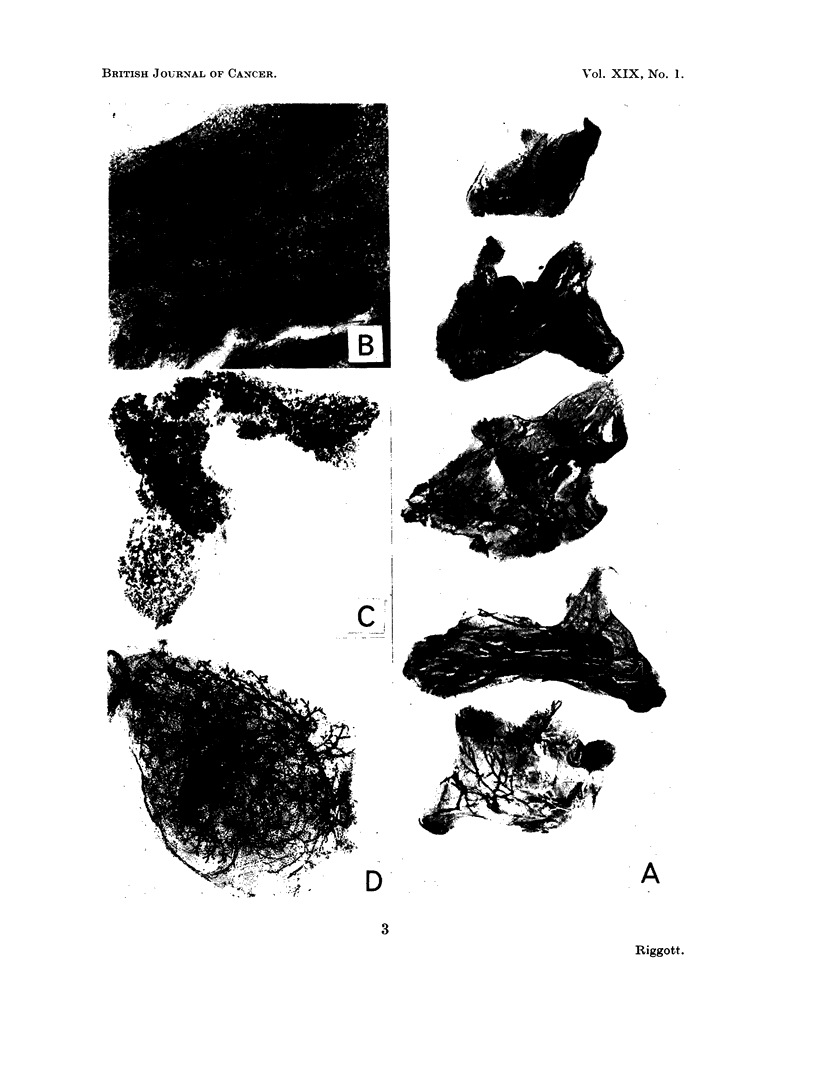

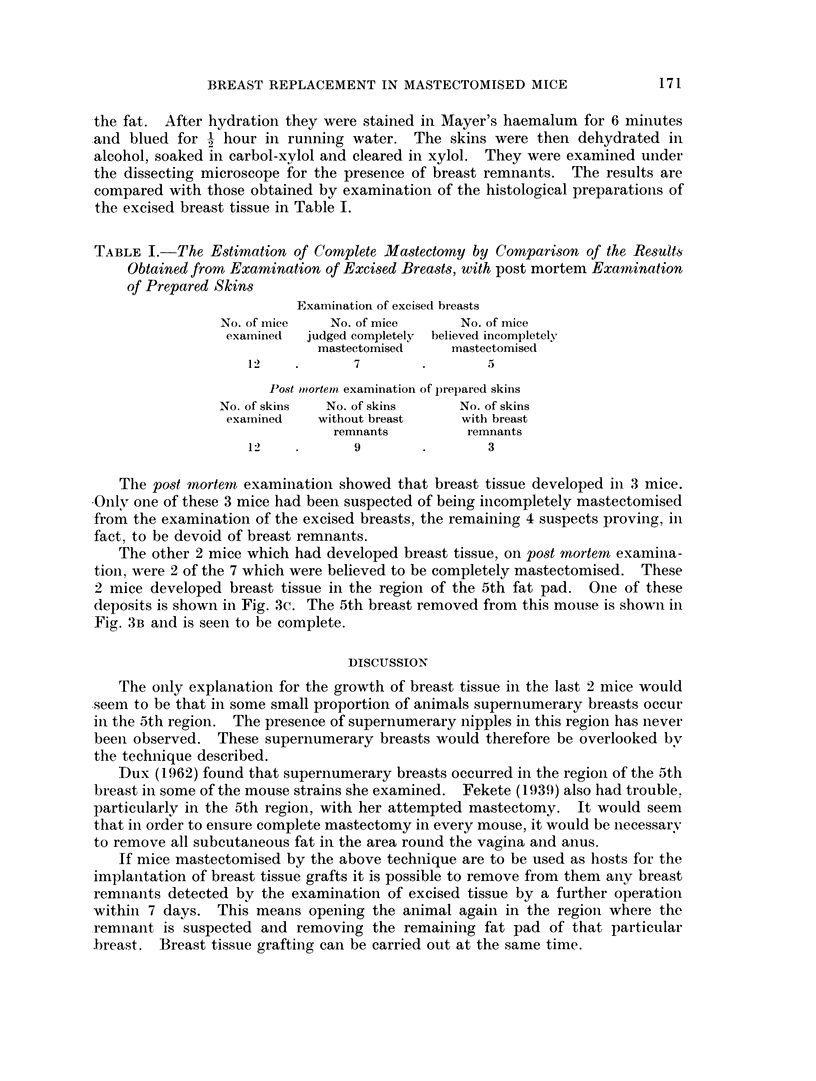

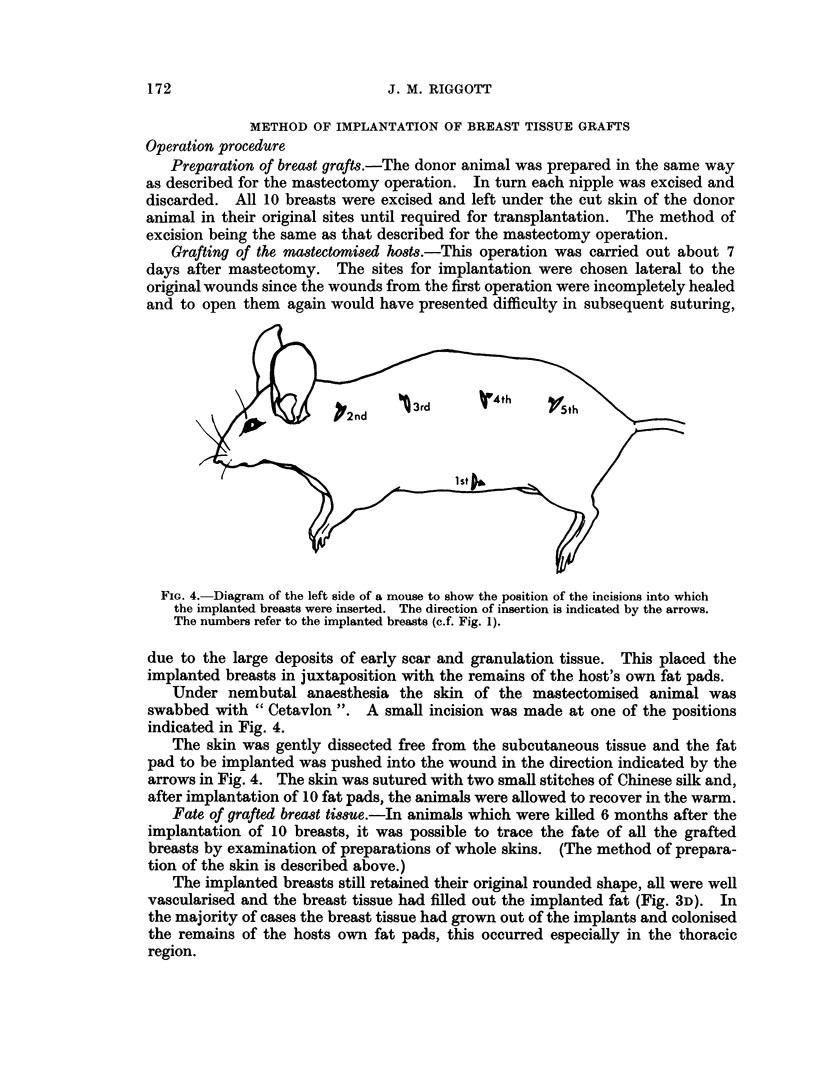

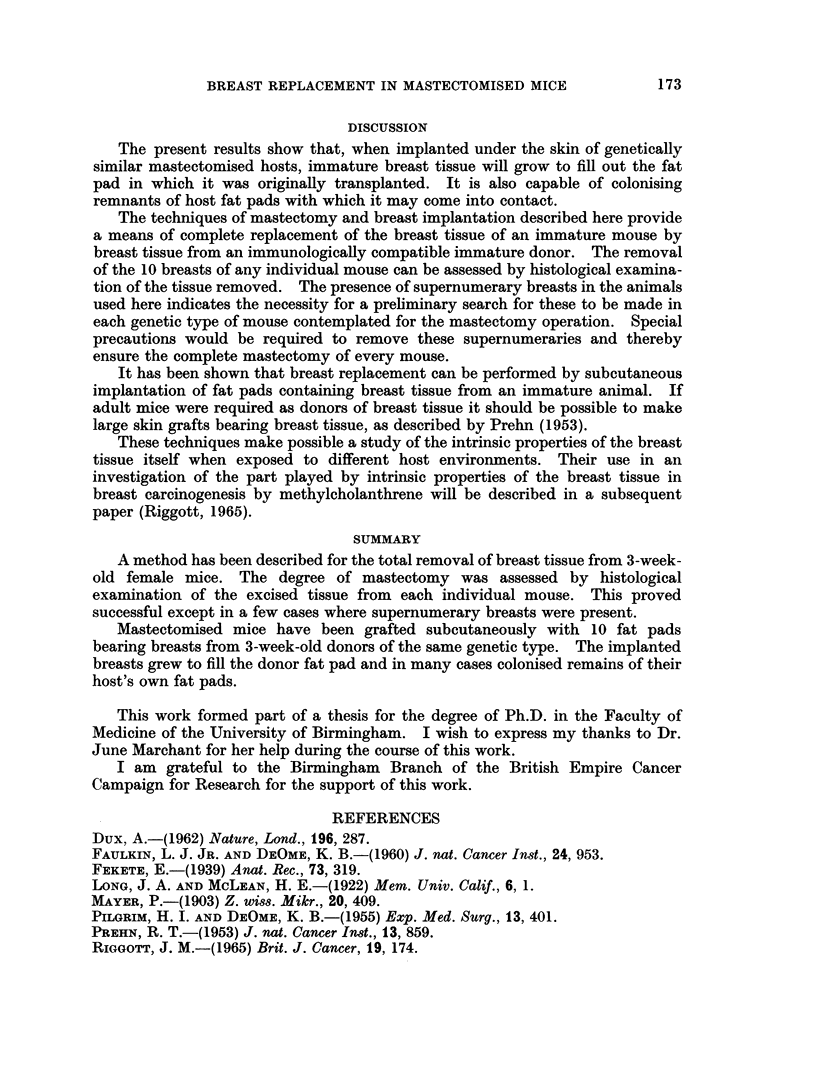


## References

[OCR_00362] FAULKIN L. J., DEOME K. B. (1960). Regulation of growth and spacing of gland elements in the mammary fat pad of the C3H mouse.. J Natl Cancer Inst.

[OCR_00368] PILGRIM H. I., DEOME K. B. (1955). Intraperitoneal pentobarbital anesthesia in mice.. Exp Med Surg.

[OCR_00370] RIGGOTT J. M. (1965). THE CHEMICAL INDUCTION OF BREAST TUMOURS IN C57BL, IF AND F1, HYBRID (C57BL X IF) BREAST TISSUE TRANSPLANTED INTO BREAST-FREE F1 HYBRID (C57BL X IF) HOSTS.. Br J Cancer.

